# Midwife-led debriefing after operative birth: four to six year follow-up of a randomised trial [ISRCTN24648614]

**DOI:** 10.1186/1741-7015-4-3

**Published:** 2006-03-01

**Authors:** Rhonda Small, Judith Lumley, Liesje Toomey

**Affiliations:** 1 Mother & Child Health Research, La Trobe University, 251 Faraday Street, Carlton Victoria 3053, Australia

## Abstract

**Background:**

There is little evidence that single-session debriefing is effective in reducing adverse mental health outcomes after trauma. Few trials have included long-term follow-up, but two also suggest possible negative effects of debriefing. We aimed to assess longer-term maternal health outcomes in a trial of midwife-led debriefing following an operative birth, given that findings at six months could not rule out a possible adverse effect of debriefing.

**Methods:**

Four to six years after participating in a midwife-led trial of debriefing following an operative birth, 1039/1041 women were mailed a questionnaire containing the Edinburgh Postnatal Depression Scale (EPDS) and the SF-36 health status measure.

**Results:**

Responses were obtained from 534 women (51.4%). Responders from the two trial groups remained comparable 4–6 years postpartum. No significant differences on maternal health outcomes were found between the trial groups.

**Conclusion:**

In the longer term, maternal health status was neither positively nor adversely affected by the experience of debriefing, despite a hint of adverse effects at six months postpartum. Short debriefing interventions have not proven effective in improving mental health outcomes for women following childbirth.

## Background

Debriefing to reduce adverse mental health outcomes after traumatic events has been in widespread use for many years. Yet evidence for the effectiveness of brief or single-session debriefing is lacking [1]. Moreover, longer-term outcomes of debriefing have been reported in only three trials (at 12 months, [2] 13 months [3] and three years [4]) and in two of these, adverse outcomes were more common in the group receiving debriefing.

Six randomised trials of debriefing have now been offered to women after birth [2,5-9]. All have focused on improving maternal mental health outcomes, recognising the potentially traumatic nature of the birth experience, at least for some women. Consistent with trials of brief debriefing interventions in other settings, four of the six trials found no benefit. Both of the two smallest trials, which showed positive effects of debriefing, reported unusually high levels of depression in the control arms (55% [6] and 32% [9]). In the latter trial [9], women were eligible for inclusion only if they reported experiencing fear for their own or their baby's life during labour or fear of serious injury or permanent damage. This selection of women with trauma symptoms might also explain the higher levels of subsequent depression. However, given the active identification of women reporting trauma symptoms, it is impossible to rule out adverse effects of assignment to a control group who were offered standard care.

We report here the four to six year outcomes of a midwife-led trial of debriefing for women who experienced an operative birth. Findings at six months postpartum demonstrated no benefit of debriefing in improving maternal mental health outcomes [5]. On the other hand – and of concern – the possibility could not be ruled out that debriefing may have contributed to emotional health problems in the intervention group. This prompted longer-term follow-up of participants.

## Methods

Ethics approval for the longer-term follow-up of trial participants was obtained from the recruiting hospital and the auspicing university ethics committees. Follow-up questionnaires were posted to 1039/1041 recruited participants. The questionnaire included the same outcome measures as the original trial questionnaire: the Edinburgh Postnatal Depression Scale (EPDS) and the self-report SF-36 health status questionnaire.

## Results

The initial mail-out of the postal questionnaire, and two reminder postcards to the contact addresses obtained at recruitment, resulted in the return of 322 completed questionnaires (31.6%). Extensive telephone follow-up of women who had not responded was then undertaken, again utilising information obtained at recruitment. Upon contact and agreement to participate, the offer of sending a second questionnaire was made. A significant minority were contactable at the same phone number, though at a new address, and so had not received the first questionnaire. Tracing of all women whose questionnaires were returned to sender, or were not contactable at the listed phone number, was also undertaken via the current electoral roll and telephone directories, and new questionnaires were sent to those women who could be traced to a new address. With specific ethics approval, we also initiated a new telephone contact procedure for identifying study participants from women with the same name but several possible new addresses. After this extensive tracing and follow-up, 534 women (51.4%) responded. Not all the women who could be contacted by telephone agreed to participate; some of those who did subsequently failed to return questionnaires. We did not re-contact these women to collect data from them by telephone, mostly because of a lack of resources. There were 264 responders from the debriefing arm and 270 from the standard care arm.

Responders in the two trial arms remained comparable in terms of status at recruitment, with no differences in mode of birth, parity, maternal age, marital status, education, income or health insurance status (Table 1). The differences reported between responders and non-responders at the six month follow-up [5] were also apparent at the longer-term follow-up; responders at 4–6 years more likely to have been married, to be older, to be better educated and to have higher family incomes at recruitment than non-responders (Table 1). They were also somewhat more likely than the non-responders to have been having their first baby at trial recruitment.

Of the women responding at four to six years, there was no significant difference between the trial arms in the proportions who had scored as depressed at six months postpartum: 35/81 (43.2%) responded from the debriefing arm and 34/65 (52.3%) from the standard care arm; OR = 0.69; 95% CI: 0.34–1.41).

Maternal health findings at four to six years are summarised in Table 2. There were no differences between the trial arms in the proportion of women who scored as probably depressed on the EPDS. Nor were there differences in mean EPDS scores. There were also no significant differences in reports that depression had been a problem for the women during the previous four weeks. Finally, there were no significant differences on the SF-36 mental health or physical health component (summary) scores.

## Conclusion

Taken together, these follow-up findings are reassuring. Debriefing after an operative birth appears to have no longer-term adverse effects on women, despite a hint of this at the initial follow-up at six months postpartum. However, no benefits of debriefing emerged in the longer-term, a finding that further reinforces the conclusion that short debriefing interventions have not proven effective in improving mental health outcomes for women following childbirth.

## Competing interests

The author(s) declare that they have no competing interests.

## Authors' contributions

RS contributed to the grant application, co-ordinated the study, conducted the data analysis and drafted the paper. JL drafted the grant application for the study, participated in protocol design and discussion of core ideas at research team meetings, and contributed to the writing of the paper. LT participated in data collection and data management processes, and contributed to the data analysis and to the writing of the paper.

## Pre-publication history

The pre-publication history for this paper can be accessed here:



## Figures and Tables

**Table 1:** Recruitment characteristics of responders* and non-responders at 4–6 years

	**Responders**		
**Characteristic (denominator: debriefing/standard care/non-responders)**	**Debriefing** ** No (%)**	**Standard Care ** **No (%)**	**Non-responders ** **No (%)**
**Method of index (operative) birth (257/264/507)**			
Elective caesarean	71 (27.6)	76 (28.8)	134 (26.4)
Emergency caesarean	89 (34.6)	82 (31.1)	165 (32.5)
Forceps	84 (32.7)	93 (35.2)	175 (34.5)
Vacuum extraction	13 (5.1)	13 (4.9)	33 (6.5)
			
**Parity (257/263/505)**			
Primiparous	159 (61.9)	172 (65.4)	304 (60.2)
Multiparous	98 (38.1)	91 (34.6)	201 (39.8)
			
**Maternal age (256/264/507)**			
<25 years	14 (5.5)	24 (9.1)	90 (17.8)
25–34 years	162 (63.3)	162 (61.4)	338 (66.7)
≥35 years	80 (31.2)	78 (29.5)	79 (15.6)
			
**Marital status (257/264/507)**			
Married	207 (80.5)	214 (81.1)	377 (74.4)
Cohabiting (de facto)	41 (16.0)	40 (15.2)	94 (18.5)
Separated, divorced, widowed	6 (2.3)	3 (1.1)	5 (1.0)
Single	3 (1.2)	7 (2.7)	31 (6.1)
			
**Secondary education (256/263/505)**			
Completed year 12	190 (74.2)	183 (69.6)	299 (59.2)
Less than year 12	66 (25.8)	80 (30.4)	163 (40.8)
			
**Tertiary education (256/264/504)**			
Degree or diploma	148 (57.8)	147 (55.7)	226 (44.9)
Other or none	108 (42.2)	117 (44.3)	278 (55.1)
			
**Family Income ($AUD) (248/254/484)**			
≤20,000	24 (9.7)	22 (8.7)	94 (19.4)
20,001–30,000	28 (11.3)	30 (11.8)	67 (13.8)
30,001–40,000	31 (12.5)	48 (18.9)	70 (14.5)
>40,000	165 (66.5)	154 (60.6)	253 (52.3)
			
**Pension or benefit main income (255/262/484)**			
Yes	28 (11.0)	24 (9.2)	98 (20.2)
No	227 (89.0)	238 (90.8)	386 (79.8)
			
**Health insurance status (257/263/506)**			
Private	100 (38.9)	91 (34.6)	146 (28.9)
Public	157 (61.1)	172 (65.4)	360 (71.1)

*N = 521; excluding 13 women who responded at 4–6 years, but did not respond at six months postpartum: 7 from the debriefing arm and 6 from the standard care arm.

**Table 2:** Maternal health outcomes at four to six years postpartum

**Outcome measure**	**Debriefing**	**Standard care**	**Statistical test**
**EPDS**			
* Probable depression (Score ≥13) *n and %*	42 15.9	45 16.7	OR = 0.94 CI: 0.58 to 1.53
* *Mean score (SD)*	6.33 (5.16)	6.77 (5.69)	t = -0.94 CI: -1.37 to 0.49
**Women's report of depression **as a problem in previous four weeks *n and %*	31 11.7	33 12.2	OR = 0.95 CI: 0.54 to 1.65
**SF-36:Mental & Physical Health**			
Mental Component Score (MCS) *Mean (SD)*	47.0 (10.7)	45.2 (10.8)	t = 0.86 CI: -1.05 to 2.69
Physical Component Score (PCS) *Mean (SD)*	49.4 (8.7)	49.2 (7.7)	t = 0.30 CI: -1.21 to 1.64
